# Mobile health delivered physical activity after mild stroke or transient ischemic attack: Is it feasible and acceptable?

**DOI:** 10.1177/17474930251315628

**Published:** 2025-01-30

**Authors:** Charlotte Thurston, Sophia Humphries, Lucian Bezuidenhout, Sverker Johansson, Lisa Holmlund, Lena von Koch, Coralie English, David Moulaee Conradsson

**Affiliations:** 1 Division of Physiotherapy, Department of Neurobiology, Care Sciences and Society, Karolinska Institutet, Stockholm, Sweden; 2 Women’s Health and Allied Health Professionals Theme, Medical Unit Allied Health Professionals, Karolinska University Hospital, Stockholm, Sweden; 3 Division of Occupational Therapy, Department of Neurobiology, Care Sciences and Society, Karolinska Institutet, Stockholm, Sweden; 4 Division of Family Medicine and Primary Care, Department of Neurobiology, Care Science and Society, Karolinska Institutet, Stockholm, Sweden; 5 Theme Heart and Vascular and Neuro, Karolinska University Hospital, Stockholm, Sweden; 6 Heart and Stroke Research Program, Hunter Medical Research Institute, Newcastle, NSW, Australia; 7 Centre of Research Excellence to Accelerate Stroke Trial Innovation and Translation, The University of Sydney, Sydney, NSW, Australia; 8 The University of Newcastle, Newcastle, NSW, Australia

**Keywords:** Physical activity, mHealth, stroke, TIA, feasibility

## Abstract

**Background and aims:**

Physical activity is a key component of secondary stroke prevention. Mobile health (mHealth) interventions show promise for enhancing post-stroke physical activity, but most studies have combined mHealth with onsite services. This study evaluated the feasibility and acceptability of a fully digitalized mHealth intervention for physical activity among individuals post-stroke or transient ischemic attack (TIA) in Sweden.

**Methods:**

In this two-arm feasibility randomized controlled trial, adults with stroke or TIA were randomized to one of the following 6-month interventions: (1) the experiment group, receiving mHealth-delivered supervised exercise (two sessions weekly during months 1 to 3, one session weekly during months 4 to 6) and behavioral change techniques for physical activity (including two individual counseling and six follow-up sessions) or (2) the control group, receiving two mHealth-delivered individual counseling and three follow-up sessions. Feasibility (reach, retention, adherence, fidelity, safety) and acceptability were assessed according to pre-specified progression criteria.

**Results:**

Of 114 participants, 105 (92%) completed the 6-month intervention and 102 (89%) completed the 12-month follow-up assessment. The intervention reached individuals from 20 of 21 Swedish regions. Sixty-eight percent of participants had a stroke (of which 96% were mild), 64% were female, and the average age was 71 years (standard deviation = 9). Ninety-five percent were born in Sweden, had a high level of education (61%), and an average daily step count of 6451 steps. Completion of outcome measures included digital questionnaires (98%), sensor-derived physical activity (92%), and blood pressure monitoring (97%). A total of 1781 supervised exercise sessions were delivered to the experiment group, with an adherence rate of 76%, and adherence to individual counseling and follow-up sessions was 96%. Ninety-five adverse events were recorded, of which 16 were related to the intervention (predominantly pain or muscle soreness) but non-serious. Overall satisfaction with the mobile app was 71%, and 76% of the experiment group believed the app could partly replicate in-person visits.

**Conclusion:**

The mHealth intervention was overall feasible and acceptable; however, there is a need to develop recruitment procedures to increase diversity of included participants regarding socioeconomic status and physical activity level, prior to a phase 3 trial.

**Trial Registration:**

ClinicalTrials.gov (NCT05111951).

## Graphical abstract

**Graphical abstract:**
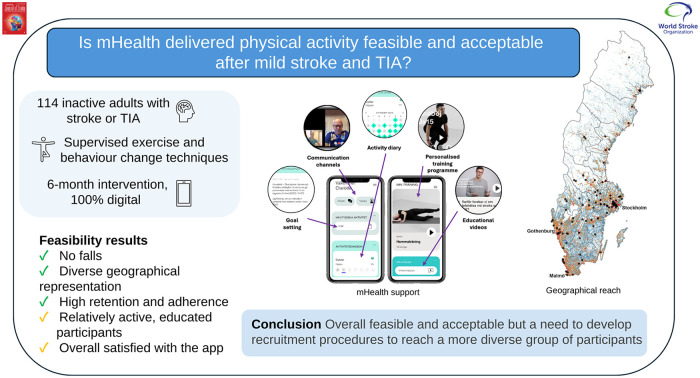

## Background

The cumulative risk of a recurrent cardiovascular event at 10 years post-stroke or transient ischemic attack (TIA) is 39.2%.^1^ Physical activity is a strong independent predictor of stroke recurrence and has been shown to reduce post-stroke disability.^2^ Despite this, low physical activity levels are prevalent, even among those who have experienced a mild stroke.^3^

Systematic review evidence suggests that physical activity interventions effective in decreasing systolic blood pressure after stroke or TIA last at least 4 months and combine supervised exercise with behavior change techniques.^4^ However, limited access to, and availability of, support for physical activity are barriers commonly encountered by individuals post-stroke and TIA.^3^ This is particularly challenging for both those living in rural areas and those with mild stroke or TIA; the latter group representing most people living with stroke, yet often lacking structured support for physical activity.

Mobile health (mHealth) (delivery of medicine and public health via mobile devices^5^) is a promising solution for reaching many individuals who are physically inactive. This expanding field ranges from information provision only to rehabilitation guidance, predominantly focused on self-management.^6^ Current low-to-moderate level evidence suggests that remote services such as mHealth are no less effective than usual care post-stroke; however, there is uncertainty around the feasibility and effectiveness of mHealth-delivered physical activity.^7^ Few studies have explored a fully digital, mHealth-supported approach to promoting physical activity post-stroke or TIA. To maximize mHealth’s reach and accessibility, the feasibility of such a strategy needs further investigation.

This study was built *upon i-REBOUND Let’s get moving*,^8^ an Australian telehealth intervention delivered via Zoom videoconferencing. *i-REBOUND* supports home-based exercise and physical activity for people with stroke or TIA and has shown to be feasible, safe, and with promising preliminary effects in Australia.^8^ Together with stakeholders, we previously adapted *i-REBOUND* into a fully digital intervention to be delivered via a mobile app, STAAR (Stroke Treatment through Active and Accessible Rehabilitation). The feasibility of the intervention in a new context (i.e. Sweden) and via a new format (i.e. mobile app) needs to be investigated prior to a phase 3 trial. The overarching aim of this study was, therefore, to assess the feasibility and acceptability of the mHealth version of the *i-REBOUND* intervention delivered across Sweden.

Research questions:

*RQ1. Did the mHealth intervention reach a representative sample of participants with stroke and TIA and to what extent were they retained during the trial?*
*RQ2. To what extent did participants adhere to the trial protocol and digital intervention sessions?*
*RQ3. Was the mHealth intervention safe?*
*RQ4. Was the mHealth technology deemed acceptable to participants?*

## Methods

### Design

This study was a two-arm feasibility randomized controlled trial of the mHealth version of *the i-REBOUND Let’s get moving* intervention. Full methodological details reported in accordance with the TIDieR checklist have been described elsewhere.^9^ The focus here is on intervention feasibility and acceptability; clinical outcomes and process evaluation will be reported elsewhere. Approval was given by the Swedish Ethical Review Authority (dnr 2020-05062, 2021-03622) and reporting follows CONSORT recommendations. All participants provided written informed consent.

### Participants and recruitment

We aimed to recruit 120 participants to provide diversity in diagnosis, disability level, and geographical location. Recruitment was administered through advertisements via the Karolinska Institutet homepage, outpatient clinics, patient organizations, and social media across Sweden. Inclusion criteria were (1) 3 months to 10 years post-stroke or TIA, (2) living at home, (3) able to walk short distances with or without a walking aid, (4) able to use a smartphone, (5) access to a stable Internet connection, and (6) able to digitally self-identify via BankID (Swedish secure self-authentication app). Exclusion criteria were (1) already meeting the recommended levels of 150-min per week of moderate or 75-min per week of vigorous intensity physical activity,^10^ (2) severe health conditions (e.g. cardiac condition), (3) significant cognitive impairment, neglect, or aphasia compromising participation in the intervention, or (4) enrolled in a concomitant clinical trial or aerobic rehabilitation.

Those reporting interest were screened for eligibility in a three-step process. First, individuals were screened via phone to assess initial trial eligibility, including physical activity level using the International Physical Activity Questionnaire.^11^ Second, a video call was conducted to evaluate the ability to follow instructions, safety awareness regarding home exercise, and smartphone proficiency. Third, eligibility was determined based on steps 1–2 and a medical certificate confirming diagnosis and capability for moderate-intensity physical activity.

### Baseline assessments

Baseline data collection included stroke or TIA history, self-reported demographics, geographical location, comorbidities, 1-year fall history, disability level (modified Rankin Scale^12^) self-perceived impact of stroke (Stroke Impact Scale^13^), and prior experience of digital health.

#### Randomization

Following baseline data collection, participants were randomized to either (1) the experimental intervention receiving the mHealth version of the *i-REBOUND* intervention or (2) the control intervention receiving only mHealth-delivered individual counseling and follow-up sessions. Randomization was conducted in blocks of two and stratified according to use of a walking aid (yes/no) and geographical location (rural/urban). Randomization was managed by an independent researcher and coded into a trial database with concealed allocation.

### Interventions

The experimental and control arm interventions lasted 6 months; full intervention details are provided elsewhere.^9^ All intervention components were conducted digitally via the STAAR app and the corresponding online clinic, using both synchronous (e.g. videoconferencing for individual counseling and supervised exercise) and asynchronous elements (e.g. chat function, educational videos, and digital questionnaires). All physiotherapists delivering the intervention were experienced in stroke rehabilitation.

#### Experiment group—mHealth version of the i-REBOUND intervention

During months 1 to 3, supervised physical exercise sessions were conducted twice weekly, and once weekly during months 4 to 6. These sessions consisted of 20 min of moderate-intensity exercise in four interval blocks, alternating between a more and less demanding exercise. Participants initially attended at least two individual exercise sessions before being eligible for group sessions (maximum four participants per group). Participants reported exercise intensity during each session using the Borg Rating of Perceived Exercise Exertion Scale^14^ to ensure at least moderate intensity was met. According to participants’ needs and desires, an individual strength and exercise program were prescribed during months 2 to 6, in the form of instructional videos.

Support for behavior change comprised of two individual counseling sessions in month 1, including exploration of motivation, exercise preferences, barriers to physical activity, and SMART goal setting (i.e. Specific, Measurable, Achievable, Realistic, Timely).^15^ Alongside this, participants where provided with access to educational videos (including physical activity principles and recommendations, exercises, and health benefits of physical activity) and an optional activity diary for self-monitoring throughout the intervention. Monthly structured follow-ups were conducted, centered around goal refinement and activity diary recordings. Participants were able to contact the physiotherapist via the chat function during the intervention period.

#### Control group—standard approach for physical activity promotion

This intervention consisted of two individual counseling sessions in month 1 and access to educational videos; both components were as per the experiment group (except for no access to exercise videos). Three structured follow-ups, one of which was optional, were provided and focused on goal progression. Communication with the physiotherapist was limited to scheduled sessions only.

### Feasibility and acceptability outcomes

Data were collected from study launch to the final 12-month follow-up.^9^ Pre-specified progression criteria were developed regarding study reach, participant retention, adherence to outcome measures, intervention fidelity and safety, and app acceptability (see Table 1). These were developed based on recommendations and similar studies^16–18^ and modified following feedback from the wider research group. The traffic light system facilitates decision-making on whether, and how, to advance to a full-scale trial.^18^ Outcomes meeting green criteria are assumed to require no further amendments; amber criteria are assumed to need a mild-to-moderate amount of modification, and any red criteria suggest major concerns about advancement to a full-scale trial. Feasibility questions without a specified value (e.g. Q.5) were discussed in the project group to reach a mutual interpretation.

**Table 1.** Progression criteria depicted by feasibility questions under study.

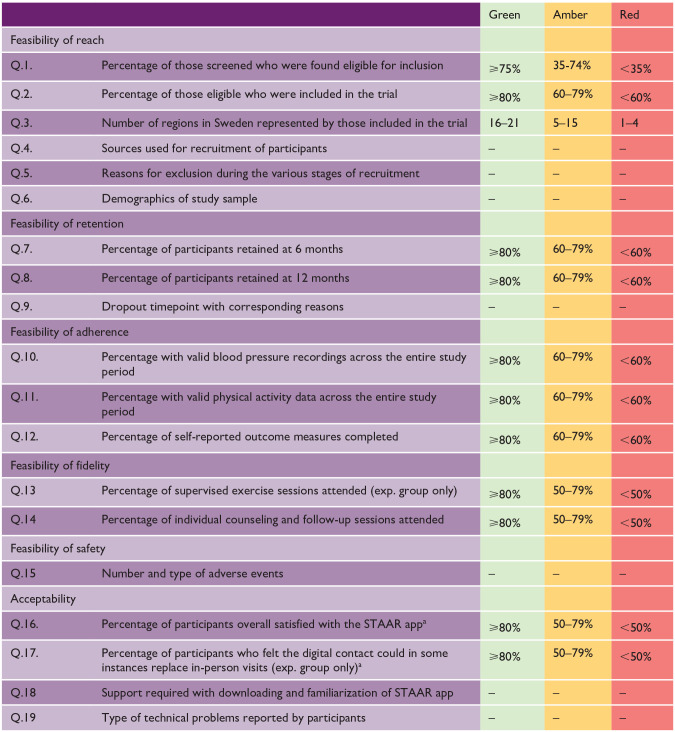

a Assessed using a modified version of the Telehealth Usability Questionnaire (TUQ).

For *reach* and *retention*, participant demographics and a flow of participant information was recorded. Geographical distribution of study participants was presented in relation to population density in Sweden.

For *adherence*, blood pressure, physical activity, and self-reported function and wellbeing were collected at baseline, and 3, 6, and 12 months after baseline assessment. Participants measured blood pressure twice daily for 7 days using an Omron M7 Intelli IT-AFIB monitor; at least six measurements over 3 days were required for assessments to be valid.^19^ Physical activity was measured using an activPAL activity monitor during the same period,^20^ with a minimum of 10 h of physical activity data per day for at least 3 days required for valid assessments. Self-reported function and wellbeing included exercise self-efficacy (Exercise Self Efficacy Scale^21^), balance confidence (Activities-Specific Balance Confidence Scale^22^), walking ability (Generic Walk-12 Scale^23^), fatigue (Fatigue Severity Scale^24^), psychosocial wellbeing (Depression Anxiety Stress Scale^25^), and health-related quality of life (EuroQol-5 Dimensions^26^). The percentage of self-reported clinical outcomes completed across all time points was calculated and used for analysis. Reminders (via SMS or chat function) were sent to participants in instances of non-completion of measures, and reasons for invalid data were recorded at each time point.

For *fidelity*, the percentage of attended supervised exercise sessions, counseling, and follow-up sessions were calculated from the total number of sessions offered. For *safety*, all adverse events (related and unrelated to the intervention) during the study period were recorded by the physiotherapists and research team during the study period. Each event was considered on an individual basis as to its severity and consequences, according to Good Clinical Practice guidance.

For *acceptability*, item 13 (“contact with the physiotherapist via the STAAR app could in some cases replace in-person visits”) and item 20 (“overall satisfied with the STAAR app”) of a modified version of the Telehealth Usability Questionnaire^27^ were used. The questionnaire was administered following intervention termination, and the items were scored on a 7-point Likert-type scale (1 = disagree, 7 = agree); score ⩾6 was interpreted as agreeing. Participants reported technical problems to the physiotherapist or via the app support function, which were subsequently analyzed and categorized. Support required for app download and familiarization was classified as minimal (one additional phone/videocall) or significant (multiple phone/videocalls).

### Analysis

Demographic data was analyzed descriptively in SPSS v29.0.2.0 and presented as number (percentage) and mean (standard deviation). Feasibility and acceptability data were analyzed descriptively and interpreted using the progression criteria (Table 1).

## Results

### Reach (Q.1–6)

**Figure 1.**
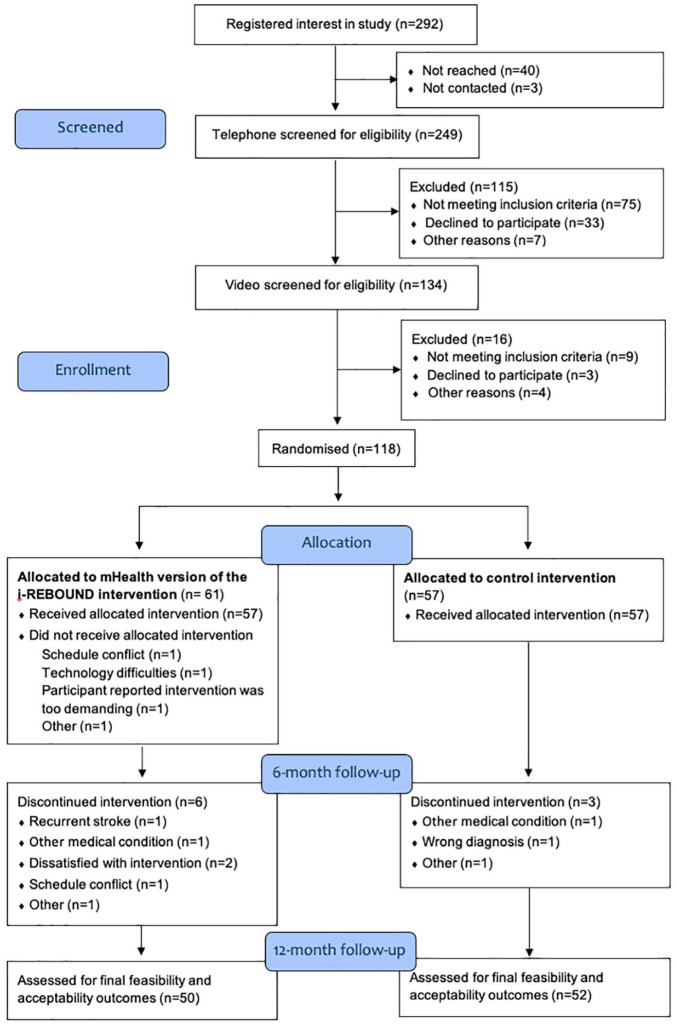
Flow of participants throughout the trial.

Screening was conducted in two periods (September to November 2021 and June to August 2022). Two hundred and ninety-two individuals registered interest in the study; 134 (46%) were eligible for inclusion, 118 (97%) were randomized, and 114 (97%) started the intervention (Figure 1). The main reason for non-inclusion was already meeting physical activity recommendations. Of the 114 participants, 68% had experienced a stroke, 64% were female, and the mean age was 71 years (range = 36–89 years, Table 2). The majority reported 0–2 on the modified Rankin Scale (n = 110, 96%), were born in Sweden (n = 108, 95%), and had a high level of education (n = 69, 61%). Around half of participants had a Fatigue Severity Scale score ⩾4, (n = 55, 48%) and baseline physical activity was an average 6451 steps per day (min–max: 43–16,251 steps).

**Table 2.** Demographic data of study participants starting the intervention.

	Experiment (n = 57)	Control (n = 57)
Age, years, mean (SD)	71.0 (8.3)	70.0 (9.5)
Female, n (%)	38 (67)	35 (61)
University/college education, n (%)	31 (54)	38 (67)
Working, n (%)	9 (16)	9 (16)
Living alone, n (%)	20 (35)	20 (35)
Born in Sweden, n (%)	54 (95)	54 (95)
Living rurally, n (%)	17 (30)	17 (30)
Stroke, n (%)	39 (68)	39 (68)
Time since stroke/TIA, years, mean (SD)	3.4 (2.9)	3.2 (2.7)
⩾3–6 months, n (%)	4 (7)	4 (7)
⩾6 months–1 year, n (%)	8 (14)	12 (21)
1–10 years, n (%)	45 (79)	41 (72)
Walking aid, n (%)	18 (32)	14 (25)
Self-reported fall in the last year, n (%)	18 (32)	19 (33)
Modified Rankin scale		
No symptoms, n (%)	9 (16)	16 (28)
No significant disability, n (%)	44 (77)	34 (59)
Slight disability, n (%)	3 (5)	4 (7)
Moderate disability, n (%)	1 (2)	2 (4)
Moderate severe disability, n (%)	0 (0)	1 (2)
Severe disability, n (%)	0 (0)	0 (0)
Fatigue (⩾4/7) on Fatigue Severity Scale, n (%)^a^	27 (47)	29 (51)
Depression, Anxiety, and Stress Scale		
Depression (⩾10/42), n (%)^a^	17 (30)	17 (30)
Anxiety (⩾8/42), n (%)^a^	11 (20)	12 (21)
Stress (⩾15/42), n (%)^a^	6 (11)	8 (14)
Stroke Impact Scale (0–100), self-perceived recovery, mean (SD)^b^	72 (24.4)	75 (23.2)
Comorbidities:		
Diabetes mellitus, n (%)	5 (9)	6 (11)
Hypertension, n (%)	39 (68)	29 (51)
Heart disease, n (%)	15 (26)	12 (21)
Joint disease, n (%)	24 (42)	20 (35)
Steps per day, mean (SD)	6889 (3431)	6013 (3366)
Daily use of mobile apps, n (%)	53 (93)	51 (90)
Digital health experience (videocall), n (%)	18 (32)	19 (33)

a Higher score denotes more severe symptoms.

b Higher score denotes higher self-perceived recovery.

**Figure 2.**
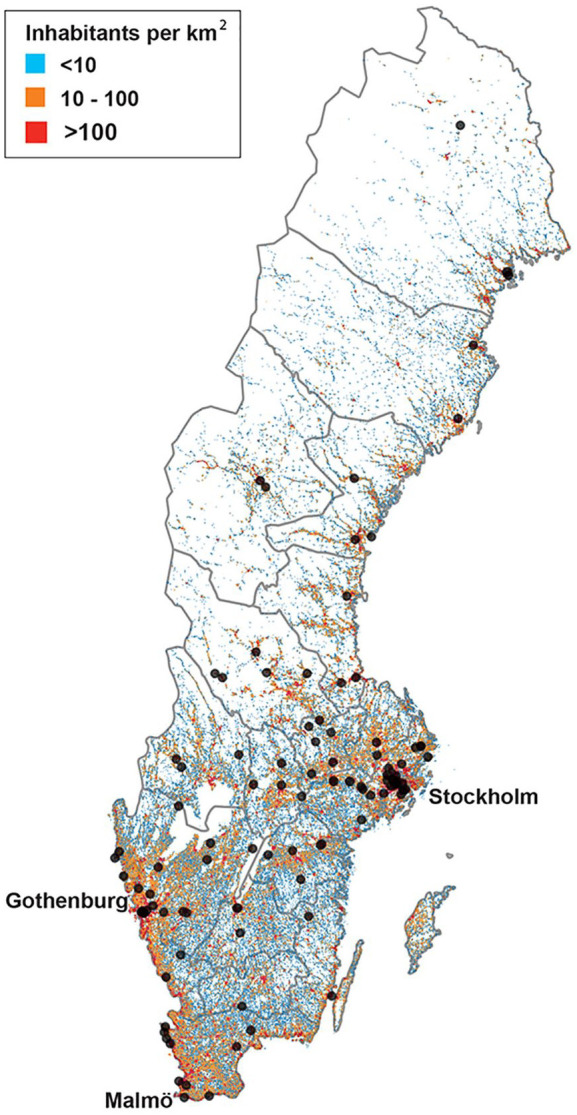
Geographical spread of participants in relation to population density of Sweden.

Participants were recruited from 20 of the 21 Swedish regions (Figure 2) with the majority living in central and southern parts of the country, where population density is the highest. Recruitment was primarily via social media (82%), followed by patient organizations (14%). Few participants were recruited through stroke rehabilitation clinics (4%).

### Retention (Q.7–9)

A retention rate of 92% (n = 105) at 6 months and 89% (n = 102) at 12 months was observed (Figure 1 and Table 3). Reasons for dropout were diverse, and the majority (8 out of 12) occurred prior to week 4 (Figure 1).

**Table 3.** Feasibility and accessibility outcomes.

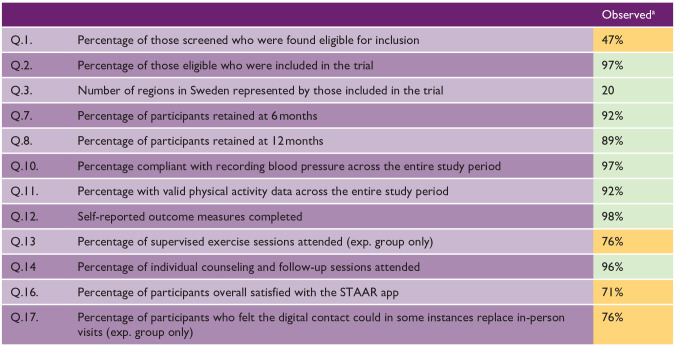

a Green = no further amendments required, Amber = mild-to-moderate amendments required.

### Adherence (Q.10–12)

Ninety-two percent of physical activity data and 97% of blood pressure data collected across the study period were valid. Likewise, 98% of questionnaires were completed. Main reasons for invalid physical activity data were device malfunction and participants declining to wear the monitor, while invalid blood pressure data related mostly to an insufficient number of recordings.

### Fidelity (Q.13 and 14)

In total, 1781 exercise sessions were delivered to the experiment group. An average of 25 sessions were attended per participant (min–max: 0–34), resulting in an attendance rate of 76%. Main reasons for non-attendance included illness, other priorities, and unspecified reasons. The attendance rate for individual counseling and follow-up sessions was 98% and 94% for the experiment and control groups, respectively. A total of 670 individual counseling and follow-up sessions were delivered. A median value of eight sessions per participant were attended in the experiment group (min–max: 0–8), and five sessions in the control group (min–max: 0-5).

### Safety (Q.15)

Ninety-five adverse events were reported during the study period. Eight events were serious, that is, resulting in death (n = 2) or hospitalization (n = 6), but were not deemed related to the physical activity components of the intervention. Sixteen adverse events were considered related to the intervention with pain or muscle soreness as the most common (n = 13). Exercise sessions were subsequently modified or canceled but did not lead to participants dropping out. No falls occurred during supervised exercise sessions.

### Acceptability (Q.16–19)

Seventy-one percent of participants were overall satisfied with the app, and 76% of the experiment group felt the app could partly replicate in-person visits. A small amount of extra support with app download and familiarization was given to 37 participants (28%), whereas 13 participants (10%) required a significant amount of extra support. Most frequently reported technical problems were related to device compatibility (e.g. error when using a tablet), followed by videoconferencing (e.g. sound quality), group supervised training (e.g. unable to join), and usability of individualized functions (e.g. activity diary errors). The main consequences of these problems included supervised training non-attendance or rescheduling.

## Discussion

This novel feasibility study used a completely digital intervention to support the provision of physical activity to individuals with predominantly mild stroke or TIA across Sweden. Overall, the intervention was found to be feasible and acceptable. Further refinement of the recruitment methods, to reach a participant group with more diverse socioeconomic status and physical activity levels, is required before commencing a phase 3 trial of effectiveness.

We found around half of those interested in study participation to be eligible for inclusion, similar to other digitally delivered trials^8^ and slightly lower than seen in non-digital intervention trials supporting physical activity.^28^ Compared with the Swedish stroke population, our cohort included more females (64% vs. 47%) and was on average 4 years younger, whereas the proportion of stroke to TIA diagnosis was similar.^29^ Most participants were Swedish-born with a mild disability, had a high level of education, were familiar using mobile apps, and relatively active at baseline. We observed an average daily step count of 6451 steps per day, greater than the 4078 daily steps reported in a systematic review on physical activity in the chronic phase post-stroke.^3^ Already active participants are not unexpected given the recruitment bias in physical activity interventions toward those who are already active and/or motivated.^30^ While age, diagnosis, and severity of disability closely match the target population, we suspect that there is a slight sociodemographic underrepresentation in terms of male participants, non-Swedish ethnicity, low education levels, and working-age individuals. This is likely due to challenges in recruiting diverse populations to digital interventions for chronic conditions.^31^ In addition, around half of our participants reported problematic fatigue, similar to that globally reported by stroke survivors.^32^ While mHealth interventions could be challenging for some with fatigue, for example, frequent screen usage required, digital interventions may be preferred due to avoidance of over-stimulating environments^33^ and a more accessible format for support for physical activity.

The retention rates at both 6 and 12 months (92% and 89%, respectively) were higher than found in other mHealth studies.^34^ The high retention rates could be explained by the interactive communication channels^35^ and support provided for behavior change,^36^ aspects which were incorporated based on earlier feedback from end-users. Adherence to clinical outcome measures was also high. The app-based format, that is, a single platform, for recording information may have contributed to this, both easing demands on participants, for example, no travel requirements, and permitting reminders to be easily sent. Conversely, several technical problems were reported and may have affected retention and adherence. However, the majority of these problems diminished over time due to close support from the app developers throughout the intervention. Despite these challenges, the overall benefits of mHealth interventions are likely to outweigh the weaknesses.^37^

Supervised exercise adherence was 76%, close to the target of 80%. Factors such as fatigue, schedule clashes for those of working age, and a repetitive exercise routine may have influenced the adherence rate.^35^ These factors are important given the increasing prevalence of strokes in younger working-age adults,^38^ highlighting the need for flexible delivery of the intervention if it is to be acceptable to a wider range of individuals. Despite these findings, adherence was still found to be higher than found in in-person physical activity interventions for stroke.^39^

Safety was of paramount importance in this study (given the absence of an onsite physiotherapist). To ensure intervention safety, comprehensive measures were adopted during both screening processes and supervised exercise sessions, including exercise modifications. As with other digital health interventions for chronic conditions,^7,40^ a number of non-serious adverse events were observed (e.g. muscle soreness); however, no serious adverse events were connected to the mHealth intervention.

### Methodological considerations

This study offers a comprehensive evaluation of feasibility and acceptability aspects of a new mHealth intervention in a large and geographically diverse Swedish sample. Our sample included a relatively homogonous participant group, and we acknowledge that recruitment predominantly via social media may have contributed to this; generalization to other stroke sub-populations should be done with caution. To increase the diversity, future studies may benefit from more active and targeted recruitment strategies such as community engagement, building relationships with clinics and redesign of the study advert. The scope of this article did not allow for full exploration of fidelity (including intervention delivery), intervention processes, or in-depth understanding of user experiences; aspects which would be beneficial to investigate prior to a full-scale trial. Finally, a cost analysis is warranted to understand the full benefits of mHealth-delivered physical activity for secondary stroke prevention.

## Conclusion

This mHealth intervention, which was found to be largely feasible and acceptable for individuals with predominantly mild stroke or TIA, shows promise for improving access to physical activity on a large scale. Recruitment strategies to reach a more diverse group of participants, increasing equity of support for physical activity, should be targeted prior to a full-scale trial.
